# Discovery of Multitarget-Directed Ligands Against Influenza A Virus From Compound Yizhihao Through a Predictive System for Compound-Protein Interactions

**DOI:** 10.3389/fcimb.2020.00016

**Published:** 2020-02-11

**Authors:** Lvjie Xu, Wen Jiang, Hao Jia, Lishu Zheng, Jianguo Xing, Ailin Liu, Guanhua Du

**Affiliations:** ^1^Institute of Materia Medica, Chinese Academy of Medical Sciences, Peking Union Medical College, Beijing, China; ^2^The Sixth Clinical Hospital of Xinjiang Medical University, Ürümqi, China; ^3^Chinese Center for Disease Control and Prevention, National Institute for Viral Disease Control and Prevention, Beijing, China; ^4^Xinjiang Institute of Materia Medica, Ürümqi, China

**Keywords:** influenza A virus, compound yizhihao, multitarget, virtual screening, *in vitro* evaluation, biological process analysis

## Abstract

Influenza A virus (IAV) is a threat to public health due to its high mutation rate and resistance to existing drugs. In this investigation, 15 targets selected from an influenza virus–host interaction network were successfully constructed as a multitarget virtual screening system for new drug discovery against IAV using Naïve Bayesian, recursive partitioning, and CDOCKER methods. The predictive accuracies of the models were evaluated using training sets and test sets. The system was then used to predict active constituents of Compound Yizhihao (CYZH), a Chinese medicinal compound used to treat influenza. Twenty-eight compounds with multitarget activities were selected for subsequent *in vitro* evaluation. Of the four compounds predicted to be active on neuraminidase (NA), chlorogenic acid, and orientin showed inhibitory activity *in vitro*. Linarin, sinensetin, cedar acid, isoliquiritigenin, sinigrin, luteolin, chlorogenic acid, orientin, epigoitrin, and rupestonic acid exhibited significant effects on TNF-α expression, which is almost consistent with predicted results. Results from a cytopathic effect (CPE) reduction assay revealed acacetin, indirubin, tryptanthrin, quercetin, luteolin, emodin, and apigenin had protective effects against wild-type strains of IAV. Quercetin, luteolin, and apigenin had good efficacy against resistant IAV strains in CPE reduction assays. Finally, with the aid of Gene Ontology biological process analysis, the potential mechanisms of CYZH action were revealed. In conclusion, a compound-protein interaction-prediction system was an efficient tool for the discovery of novel compounds against influenza, and the findings from CYZH provide important information for its usage and development.

## Introduction

Influenza (flu) is an acute respiratory viral infection responsible for seasonal pandemics, causing up to millions of cases of severe illness around the globe each year. The influenza A virus (IAV) presents the strongest infectivity among the influenza types A, B, and C (Nicholls, 2006). The IAV is highly variable due to the constant production of unique viral strains; this occurs through genetic mutation, leading to evasion of the human immune system, which causes great difficulty in studying antiviral drugs to treat flu. There are several types of anti-flu drugs available, including inhibitors of neuraminidase (NA), the M2 ion channel, and RNA-dependent RNA polymerase (RdRp), however, their clinical use is occasionally impeded by high levels of resistance in mutated viral strains (Zu et al., 2015; Schaduangrat et al., 2016). These targets are prone to resistance in the clinic, therefore, the development of antiviral drugs with novel modes of action are of high importance.

Chinese herbal formulas have been commonly used to treat flu since ancient times and are well-developed for clinical use. Compound Yizhihao (CYZH) is a traditional Uyghur medicinal formula, consisting of *Radix isatidis, Folium isatidis*, and *Artemisia rupestris*, which is recorded in the Complication of National Standard for Traditional Chinese Medicine for treating fever, sore throat and cold symptoms. The previous study has demonstrated that CYZH had broad-spectrum antiviral effects against influenza A (H1N1, H3N2, oseltamivir resistant H1N1, amantadine resistant H3N2) and B strains *in vitro* (Yin et al., 2017). Although CYZH possesses effect against influenza virus, its active ingredients and mechanisms have not yet been elucidated. The compositions from traditional Chinese medicines (TCMs) are very complicated, and the identification of compound-protein interactions (CPIs) remains a costly and time-consuming step for biological experiments. Therefore, *in silico* prediction tools for exploring compound-protein interactions and biochemical mechanisms need to be developed.

Structure-based and ligand-based methods, such as pharmacophore modeling studies, similarity searches, and docking, are extensively used (Zhang et al., 2017). Molecular docking is a basic structure-based method for computationally exploring CPIs and estimating their binding energies. Lai et al. docked the components from anti-flu TCMs to several viral proteins to study their binding modes (Gu et al., 2013). However, molecular docking simulations are often limited by slow computational speeds and unavailable target crystallographic structures. Quantitative structure-activity relationship (QSAR) methods are of major importance for the prediction of biological activity. Liu et al. built a QSAR classification model using a support-vector machine (SVM) and Naïve Bayesian (NB) model to find NA inhibitors (Lian et al., 2016). In the supervised machine learning methods of SVM and NB, the molecular descriptors improved the predictive power of QSAR classification modeling and reduced the computational complexity by removing uncorrelated descriptors. Notably, molecular docking is still a suitable method when target inhibitor data is insufficient to build datasets for machine learning models.

A principal step in the construction of virtual screening (VS) models is in choosing target proteins responsible for pathogenesis. Single-target research is encountering bottlenecks for some complex diseases and their drugs, causing high costs and low success rates, therefore multitarget-directed ligands are an increasingly popular strategy to combat complex diseases such as cancers and neurodegenerative diseases (Benek et al., 2016; Fang et al., 2018). As the flu virus is an intracellular pathogen, the role of host factors is critical to the functioning of flu viral proteins. Targeting factors within the network of viral component-host factor interactions could be a promising way to discover novel antiviral agents (Tripathi et al., 2015; Watanabe and Kawaoka, 2015).

**Figure 1:**
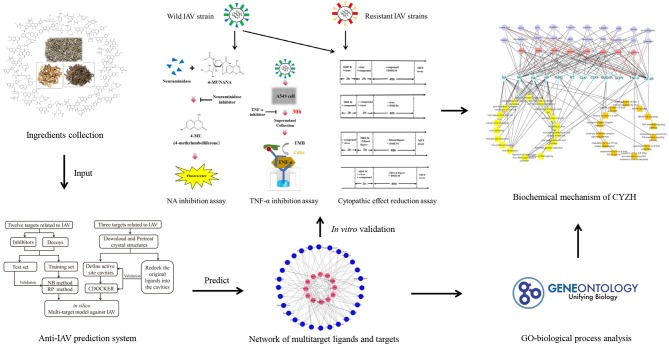
Scheme for model construction, identification of potential anti-influenza ingredients, and elucidation of the mechanisms of CYZH, based on network pharmacology approaches.

In this study, multiple key targets from the network of IAV–host interactions were investigated, including viral and host proteins. For NB, recursive partitioning (RP), and CDOCKER methods, a multitarget vs. system for CPI against the IAV was established. We applied it to predict potential targets from CYZH constituents. The most promising constituents were then validated by *in vitro* experiments. Lastly, combined with an analysis of network pharmacology, the mechanism of this drug formula was elaborated. A workflow for the integrated method is shown in Figure 1.

## Materials and Methods

### Data Collection and Preparation

Flu targets were collected from the Thomson Reuters Integrity Database (https://integrity.clarivate.com), and the supplementary targets of new drugs that had entered into at least phase I clinical trials were explored using the Therapeutic Target Database (https://db.idrblab.org/ttd/). Chemical and pharmacological information on active ligands for the collected targets was obtained using the Binding Database (www.bindingdb.org). The data sets of the ligands were refined using the following criteria: compounds were omitted if IC_50_ > 10 μM; duplicate structures were removed; SMARTS rules were used for filtering molecules carrying a charge and salts that are converted into acids or bases. Decoy compounds were generated using the DUD-E (http://dude.docking.org/) online tool. For each target, both active compounds and triple amounts of decoy compounds were randomly but proportionally divided into training sets and test sets; the ratio of training set vs. test set was 3:1. For the targets whose ligands' number was <50, molecular docking was considered as the better choice for target identification. The crystallographic structures of targets obtained from the RCSB Protein Data Bank (http://www.rcsb.org/) were imported into Discovery Studio 2016 (Accelrys Software, Inc., San Diego, CA, USA) for use in the docking method. Protein preparation was carried out based on the following criteria: missing hydrogen atoms and missing residues were corrected, water molecules and the complexes bound to receptor molecules were removed, and energy values of proteins were minimized (Fang et al., 2014).

The reported constituents of CYZH (*Radix isatidis, Folium isatidis, Artemisia rupestris*) were obtained from the TCMSP database (http://lsp.nwsuaf.edu.cn/tcmsp.php), Drugbank (https://www.drugbank.ca/), and PubChem Compound database (https://www.ncbi.nlm.nih.gov/pccompound). After deduplication, ingredients were sorted for further prediction.

### Computational Methods and Prediction System

**Figure 2:**
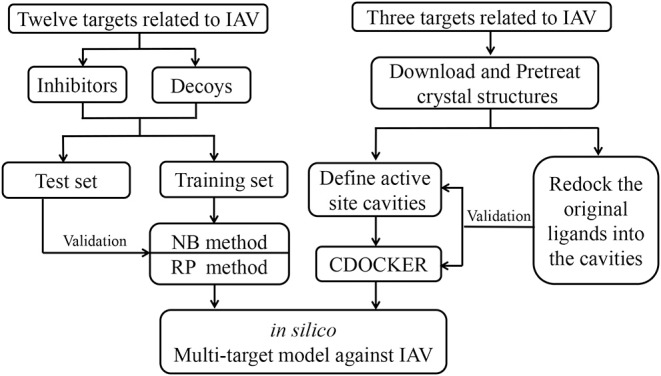
The workflow for the construction of multi-target models against IAV.

The predictive modeling system integrated both ligand-based and structure-based algorithms, including machine learning methods that use NB and RP algorithms, and CDOCKER for the remaining targets without enough inhibitors in Discovery Studio. The model building process is shown in Figure 2.

#### Naïve Bayesian

NB classifiers is a statistical approach used for categorization, which is based on the frequency of the occurrence of features (molecular fingerprints and properties) that can distinguish differences between active and decoy compounds. It generates posterior probability from *a priori* probability and data based on the core of the function, as Equation (1) shows. A relative predictor can process large datasets with fast learning ability, be tolerant of random noise, and estimate the likelihood of data samples with activity.

(1) $$\begin{array}{l} P\left(Y|X\right)=\frac{P\left(X|Y\right)\;P\left(Y\right)}{P\left(X\right)} \end{array}$$

Here, X and Y are independent events. P(X) is the marginal probability of the given molecules that will occur in the dataset; P(Y) is the *a priori* probability induced from a set of compounds in the dataset; P(X|Y) is the conditional probability that a particular molecule is classified as being bioactive in the dataset.

Extended connectivity fingerprints (ECFPs) are circular topological fingerprints that have the advantage of being rapidly calculated, and present stereochemical information and chemical substructures. As fragments should be neither too large nor too small, the diameter of 6, namely, an ECFP_6 fingerprint, was selected for each fingerprint (Fang et al., 2013). Hence, ECFP_6 fingerprint and default molecular descriptors were used for small molecular description in building NB classifier models.

#### Recursive Partitioning

RP is a classification method used to recursively partition a group of compounds into smaller and smaller subsets by a set of hierarchical rules until the active response variable becomes homogeneous. As a result, compound groups are classified into similar response nodes. The developed RP model is featured by “decision tree,” which can reveal the relationship between a dependent property (activity class) and independent properties (molecular fingerprints and properties), so it can be used to categorize samples into active and decoy compounds (Chen et al., 2011; Fang et al., 2015). ECFP_6 fingerprint and default molecular descriptors were also used as chemical descriptors.

#### Performance Evaluation of Models

Five-fold cross-validation and test set validation methods were used to evaluate NB and RP classifiers. In the 5-fold cross-validation, the dataset was randomly partitioned into five equal-sized splits. The model was trained on four out of the five cross-validation splits, and the fifth subset was used to assess the model. For both validation methods, the Matthews correlation coefficient (MCC) and the area under the receiver operating characteristic curve (AUC) were considered as the vital index. The MCC can be used to represent the quality of a binary classifier system, which varies a value between −1 and +1. A value of +1 represents no difference between a predicted and observed result, 0 represents a random result, and −1 represents a definite difference between a predicted and observed result. An ROC curve is plotted to characterize the diagnostic ability of binary classification, while the AUC is calculated to quantify the ability, falling in the range of 0–1 (Fang et al., 2015). The greater the score, the better the performance, with the perfect classification giving an AUC value of 1, an uninformative classifier yielding 0.5, whereas 0 represents no performance.

#### CDOCKER

Molecular docking, based on the 3D structure of biological proteins obtained by X-ray diffraction, nuclear magnetic resonance data, or homology modeling, is a process that identifies the complementary molecules for a target spatially and electrically. The CDOCKER module is a CHARMm-based docking algorithm (Bhuvanendran et al., 2019), offering full ligand flexibly for high-accuracy docking results of the potential binding mode between ligands and receptors.

To determine the reliability of docking data, co-crystallized ligands were firstly re-docked into defined cavities with the CDOCKER protocol. The root-mean-square deviation (RMSD) values were then calculated between the docking and initial conformations (Pang et al., 2018). Generally, the smaller the RMSD value, the better the docking pose has to the ligand binding mode.

### Gene Ontology Biological Process Analysis

Clustering of Gene Ontology (GO) terms is a useful online tool to systematically extract the biological functions shared within a list of genes, which are rich annotations of biological process (BP), cellular component (CC), and molecular function (MF) terms represented by GO terms (Tiirikka et al., 2014). GO-BP annotations are based on specific and traceable scientific evidence and provide information about the biological pathways a gene product's activity contributes toward.

### Viral Strains, Cell Lines, and Reagents

Influenza virus strain A/Puerto Rico/8/34 (wild-type H1N1), A/Minfang/151/2000 (wild-type H3N2), A/HebeiXinhua/SWL1106/2017 (oseltamivir- and amantadine-resistant H1N1), and A/FujianXinluo/SWL2457/2014 (amantadine-resistant H1N1) were obtained from the Institute for Viral Diseases Control and Prevention, Chinese Center for Disease Control and Prevention. The viruses were propagated in 9-day-old embryonated chicken eggs at 35°C for 48 h and aliquoted before storing at −80°C. Their hemagglutination titers reached 1:2048, 1:32, 1:256, and 1:64, respectively.

Madin-Darby Canine Kidney (MDCK) cells and human lung cancer cell line (A549) cells were purchased from Cell Center, Institute of Basic Medical Research, Chinese Academy of Medical Sciences. MDCK and A549 cells were cultured in Dulbecco's Modified Eagle medium (DMEM) and RPMI 1640 medium, respectively, with 10% fetal bovine serum at 5% CO_2_, 90% relative humidity and 37°C.

Human TNF-α ELISA kits (Cat No.EH009) were purchased from ExCell Bio, Shanghai, China. The NA substrate 4-methylumbelliferyl-a-D-N-acetylneuraminic acid sodium salt hydrate solution (MUNANA, Lot#M8639), thiazolyl blue tetrazolium bromide (MTT, Lot#M5655), and TPCK-treated trypsin (Lot#SLBW1439), were purchased from Sigma Aldrich.

The potential active constituents from CYZH were purchased from Sichuan Wei Keqi Biological Technology Co., Ltd., and Shanghai source Leaf Biological Technology Co., Ltd. Zanamivir (Lot#1724088), ribavirin with 98% purity (Lot#020M4003), and oseltamivir phosphate (Lot#BP903) were purchased from Sigma Aldrich. Stock solutions of CYZH compounds, ribavirin, and oseltamivir phosphate were dissolved in 100 mM in dimethyl sulfoxide (DMSO). These solutions were diluted to the indicated concentration for each assay.

### Neuraminidase Inhibition Assay

The neuraminidase (NA) inhibition assay (Li et al., 2015) was carried out in 96-well plates. A/PR/8/34 (H1N1), A/minfang/151/2000 (H3N2), A/HebeiXinhua/SWL1106/2017 (oseltamivir- and amantadine-resistant H1N1), and A/FujianXinluo/SWL2457/2014 (amantadine-resistant H1N1) were used as NA sources. Reaction mixtures containing 30 μL NA with either 10 μL of four serial compound sample dilutions (sample wells), 10 μl zanamivir, or water (model wells), were mixed by vibration for 1 min. In addition, blank wells were prepared using 40 μL water. Subsequently, 60 μL of the fluorescent substrate MUNANA was added to give a total of 100 μL in each reaction buffer solution. After this step, the final concentrations of the compound in sample wells were at 0.8, 4, 20, and 100 μM. After mixing by vibration for 1 min and incubating for 60 min at 37°C, 150 μL NaOH solution (34 mM) was added to terminate the reaction. The fluorescence was measured with an excitation wavelength at 360 nm and emission wavelength at 450 nm. Fluorescence values were used to calculate the inhibition rate (%) by the following Equation (2), the IC_50_, the concentration of inhibitor required to produce 50% inhibition of an enzymatic reaction at a specific substrate concentration, was calculated by percent inhibition with corresponding inhibitor concentration. Each measurement was performed in triplicate.

(2) $$\begin{array}{l} Inhibition\;rate\;\left(\%\right)=\frac{RFU\;model\;-\;RFU\;sample}{RFU\;model\;-\;RFU\;blank} \end{array}$$

Here, RFU_model_, RFU_sample_, and RFU_blank_ represent the fluorescence values of model wells, sample wells, and blank wells, respectively.

### TNF-α Inhibition Assay

A549 cells were seeded into a single layer on 96-well plates. The cells were washed, then treated with compound samples (100 μM) and the H1N1 virus A/PR/8/34 (100TCID_50_) simultaneously. In addition, control wells were prepared with serum-free medium and model wells with virus. The 96-well plates were incubated at 37°C and 5% CO_2_ for 30 h, then cell culture supernatants were collected. The levels of expressed TNF-α were detected in cell culture supernatant samples by enzyme-linked immunosorbent assay (ELISA) according to the manufacturer's protocol. Each measurement was performed in triplicate.

### Cytotoxicity Test

MDCK cells were plated in 96-well plates and incubated at 37°C in a humidified 5% CO_2_ atmosphere until reaching 80–90% confluence. After being washed, drug group cells were treated with the 28 compounds at 100 μM and negative control group cells were treated with mock control solutions, then returned to the incubator for 48 h. One hundred microliters of MTT (0.5 mg/ml) was then added to each well and cells were incubated for 4 h. Crystallized formazan in plates was dissolved in DMSO (100 μL/well) and absorbance was measured at 570 nm by spectrophotometry using a microplate reader (Molecular Devices, USA) (Ding et al., 2017). Each measurement was performed in triplicate.

### Cytopathic Effect Reduction Assay

MDCK cells were seeded on 96-well plates and grown until a 100% confluent monolayer was formed. To evaluate drug activity, two wild and two resistant type A virus strains were used in four different modes as follows: (1) Drug administration after viral infection: test samples with 6 two-fold serial dilutions (3.125, 6.25, 12.5, 25, 50, 100 μM) from the stock solutions were added to the cells 2 h after the adsorption of flu virus (100TCID_50_). (2) Viral infection after drug administration: cells were incubated with 6 dilutions of the test drugs for 2 h, then infected with flu virus at 100TCID_50_ for 2 h. (3) Pre-incubation of virus and drug: 100TCID_50_ of flu virus was pre-incubated with the six dilutions of the test drugs for 2 h before being added to the cells. (4) Simultaneous viral infection and drug administration: Cells were treated with the six serial dilutions of the test samples and flu virus (100TCID_50_) simultaneously for 2 h. In addition, control wells were prepared with serum-free medium and model wells were infected with virus for 2 h on each plate. After removal of the virus solution, a maintenance solution containing TPCK-treated trypsin that cleaves hemagglutinin (HA) was added. All plates were incubated at 37°C and 5% CO_2_ for 48 h. Cell viability was determined by MTT assay, as described in the preceding section. Each measurement was performed in triplicate. Absorbance values were used to calculate the inhibition rate (%) using the following Equation (3):

(3) $$\begin{array}{l} Inhibition\;rate\;\left(\%\right)=\frac{ODsample-ODmodel}{ODcontrol-ODmodel} \end{array}$$

OD_control_, OD_model_, and OD_sample_ mean the optical density values of control, model and sample wells, respectively. Each determination was performed in duplicate (Li et al., 2005).

### Statistical Analysis

All values are expressed as the mean ± SD from at least three independent experiments. Statistical significance was evaluated by one-way ANOVA. Differences are considered to be significant at *p < 0.05, **p < 0.01, and ***p < 0.001.

## Results

### Data Collection for Targets

Flu is a multifaceted disease with many symptoms, including coughing, runny nose, fever, headache, and pneumonia, which are related to host respiratory, nervous, and immune systems. Fifteen targets from the network of IAV–host interactions were selected (Table 1). IAV contains a genome composed of 8 RNA segments encoding the following: surface proteins HA, NA, and M2 ion channel (M2); matrix protein 1 (M1) situated beneath the membrane; three subunits of RdRp known as polymerase basic protein 1 (PB1), polymerase basic protein 2 (PB2) and polymerase acidic protein (PA); nucleocapsid protein (NP) that coats the viral genome; non-structural protein-1 (NS1); and nuclear export protein (NEP/NS2) (Loregian et al., 2014; Brooke, 2017).

**Table 1:** The name and classification of flu targets.

**Name**	**Abbreviation**	**Classification**	**Modeling method**
Hemagglutinin	HA	Viral target	CDOCKER
Nucleoprotein	NP	Viral target	CDOCKER
Matrix protein 2	M2	Viral target	CDOCKER
Neuraminidase	NA	Viral target	NB & RP
RNA-directed RNA polymerase	RdRp	Viral target	NB & RP
Reverse transcriptase	RT	Viral target	NB & RP
Cdc2-like kninase 1	CLK1	Host cellular target assisting viral replication	NB & RP
Cdc2-like kninase 4	CLK4	Host cellular target assisting viral replication	NB & RP
Opioid receptor	OPR	Neuroendocrine immunomodulation-related target	NB & RP
Dopamine receptor	D_2_R	Neuroendocrine immunomodulation-related target	NB & RP
N-methyl-D-aspartate receptor	NMDAR	Neuroendocrine immunomodulation-related target	NB & RP
Glutamate carboxypeptidase II	GCPII	Neuroendocrine immunomodulation-related target	NB & RP
Corticosteroid 11-beta-dehydrogenase	HSD11B1	Neuroendocrine immunomodulation-related target	NB & RP
Tumor necrosis factor alpha	TNF-α	Neuroendocrine immunomodulation-related target	NB & RP
Nuclear factor of kappa B	NF-κB	Neuroendocrine immunomodulation-related target	NB & RP

In addition to viral factors, the host's cellular machinery is utilized during each step of the IAV infection cycle. Cdc2-like kinase 1 (CLK1), which regulates alternative splicing of the M2 gene, was found to be a vital host factor for flu in previous research conducted by our group. Similarly, CLK4 is another key host CLK family isoform for IAV infection (Zu et al., 2015). Moreover, the neuroendocrine immunomodulation (NIM) network plays a critical role in the process of immune and infectious diseases via homeostasis and defense against outside pathogens (Wang et al., 2016). When the host innate and adaptive immune system are induced by an invading virus, a variety of cellular signal pathways are triggered. Earlier findings have indicated that NF-κB and TNF-α pathways can be activated to regulate cytokine and chemokine expression, maintaining host defense responses to the flu virus (Pinto et al., 2011; DeBerge et al., 2014). A phase 2 antiviral agent, ATL101, targets glutamate carboxypeptidase II (GCPII), which belongs to the TNF-α signaling pathway. Glycyrrhizin is a natural product that has entered into phase III clinical trials and acts on corticosteroid 11-beta-dehydrogenase isozyme 1 (HSD11B1) in the IL1 signaling pathway (Southan et al., 2016). The opioid receptor (OPR) is a widely distributed receptor found on neurons, immune cells, and epithelial cells of the oral and respiratory tract. Evidence suggests that the OPR has a functional role in inflammation and respiratory viral disease (Yan et al., 2015). The dopamine receptor (D_2_R) and N-methyl-D-aspartate receptor (NMDAR) are localized on CNS neurons and are related to neurological defects caused by IAV, which have been documented on the Thomson Reuters Integrity database.

### Data Set Analysis and Model Evaluation

#### Chemical Space Diversity Analysis of mt-QSAR Models

Generally, the predictive accuracy of mt-QSAR classification models is greatly influenced by the chemical space diversity of datasets. A classification model with a narrow chemical space usually results in its limited application. Based on this consideration, the statistical data was organized into training sets and test sets for each target (Table 2). The Tanimoto similarity index (TSI) can be used for measurements of chemical spatial regions; the smaller the TSI value, the greater the diversity of a dataset. Based on results, the TSI value ranges from 0.084 to 0.117, which indicates that data sets are sufficiently diverse.

**Table 2:** The detailed statistical description of data sets for flu targets.

**Target**	**Training set**				**Test set**			
	**Inhibitors**	**Decoys**	**Total**	**TSI**	**Inhibitors**	**Decoys**	**Total**	**TSI**
NA	160	484	644	0.117	54	162	216	0.116
RdRp	166	498	664	0.111	55	165	220	0.113
RT	172	516	688	0.1	57	171	228	0.105
CLK1	126	378	504	0.1	42	126	168	0.097
CLK4	88	264	352	0.112	29	87	116	0.111
OPR	425	1,275	1,700	0.089	142	426	568	0.087
D_2_R	176	528	704	0.092	59	177	236	0.096
NMDAR	41	123	164	0.112	13	39	52	0.103
GCPII	118	354	472	0.108	39	117	156	0.108
HSD11B1	1,900	5,700	7,600	0.096	633	1,899	2,532	0.097
TNF-α	878	2,634	3,512	0.102	293	879	1,172	0.098
NF-κB	848	2,544	3,392	0.084	282	846	1,128	0.086

#### Performance Evaluation of mt-QSAR Models

Five-fold cross-validations utilizing the training set were performed to avoid overfitting of the model. Subsequently, the generated models were used to predict respective test sets. Validation results are provided in Table 3. The 5-fold cross-validation results of the training set for the 24 classification models show that MCC values range from 0.8 to 1, with an average of 0.936, whereas the AUC values range from 0.969 to 1, with an average of 0.989, suggesting that the 24 classifiers are of high quality. However, five-fold cross-validations cannot completely represent the true predictive ability of the models, therefore test set validation was further explored. Among the 24 models, 23 models gave an MCC value >0.6; MCC values ranged from 0.327 to 1, with an average of 0.851. Twenty-three models out of 24 give an AUC value >0.9; AUC values ranged from 0.779 to 1, with an average of 0.970. This data indicates that the predictive abilities of these models are sufficient for further compound activity prediction.

**Table 3:** The 5-fold validation and test set validation performance of 12 flu targets using NB and RP classifiers.

**Target**	**5-fold cross validation**				**Test set Validation**			
	**NB**		**RP**		**NB**		**RP**	
	**MCC**	**AUC**	**MCC**	**AUC**	**MCC**	**AUC**	**MCC**	**AUC**
NA	1.000	1.000	0.967	0.981	0.977	1.000	0.965	0.917
RdRp	0.992	0.995	0.960	0.991	0.915	1.000	0.940	0.973
RT	0.992	0.996	0.800	0.969	0.891	0.993	0.327	0.779
CLK1	0.974	0.990	0.854	0.981	0.954	0.993	0.769	0.917
CLK4	0.992	0.999	0.954	0.978	1.000	1.000	0.809	0.961
OPR	0.983	0.998	0.901	0.994	0.995	1.000	0.868	0.983
D_2_R	0.989	0.997	0.895	0.994	0.978	1.000	0.868	0.978
NMDAR	0.984	0.999	0.884	0.994	0.648	1.000	0.648	1.000
GCPII	0.994	1.000	0.919	0.982	1.000	1.000	1.000	1.000
HSD11B1	0.983	0.994	0.911	0.992	0.973	0.998	0.868	0.974
TNF-α	0.949	0.981	0.802	0.973	0.846	0.988	0.663	0.923
NF-κB	0.940	0.989	0.834	0.981	0.835	0.990	0.685	0.917

#### Validation of CDOCKER Models

**Figure 3:**
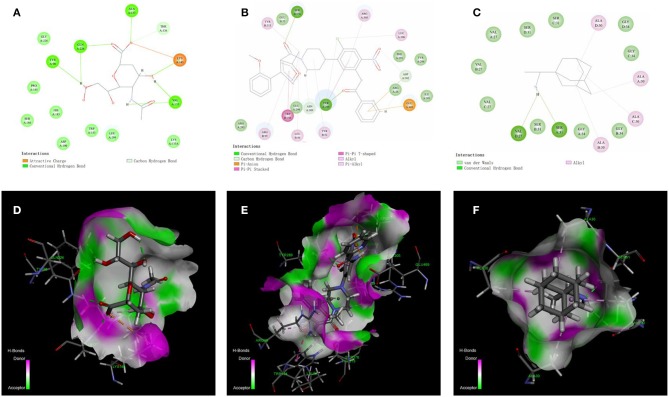
Diagrams of the non-bonded interactions between the target protein and the co-crystallized ligands. **(A,D)** show the interaction between HA and o-sialic acid. N- [4-chloranyl-5- [4- [ [3-(2-methoxyphenyl)-5-methyl-1,2-oxazol-4-yl] carbonyl] piperazin-1-yl]−2-nitro-phenyl] pyridine-2-carboxamide shows powerful interactions with NP mainly through π-π T-shaped, π-π stacked, alkyl, and hydrogen bond interactions in **(B,E)**. The non-bonded interactions between M2 and rimantadine **(C,F)** were strong with the key amino acid residues VAL27 and SER31.

The docking models for HA, NP, and M2 were constructed using the CDOCKER instead of the QSAR method due to a lack of inhibitors. The X-ray crystal structures of HA, NP and M2 were downloaded using PDB IDs for 3UBE, 4DYN, and 6BKL, respectively. Firstly, co-crystallized ligands were redocked into cavities using the CDOCKER algorithm. The lowest values for CDOCKER energy (CE) for HA, NP and M2 were −31.98, −21.9, and −6.45 kcal/mol, respectively, whereas the corresponding RMSD values were 0.73, 1.16, and 2.0 Å, respectively, which suggests that the CDOCKER models with the defined cavities were suitable for prediction. The non-bonded interactions between receptors and the most stable poses of co-crystallized ligands are shown in Figure 3.

#### Prediction of the Active Constituents From CYZH

**Figure 4:**
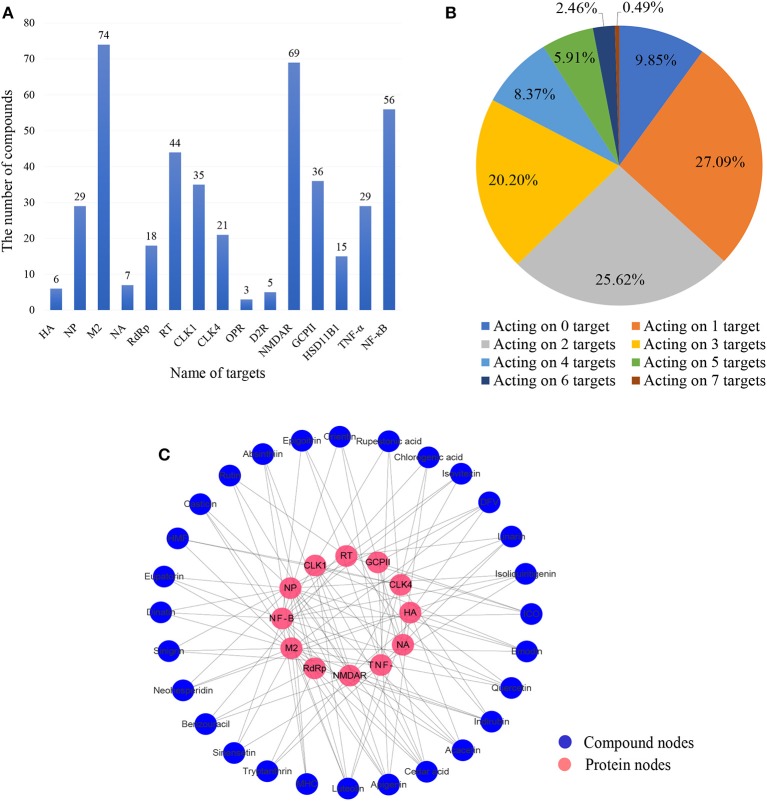
**(A)** Number of compounds corresponding to each target. **(B)** Proportion of compounds acting on different numbers of targets. **(C)** The network of 28 multitarget-directed ligands in CYZH and targets based on the prediction system against IAV. Blue circles represent drug nodes and red circles represent protein nodes.

To explore the interactions between CYZH ingredients and the 15 anti-IAV targets, predictions were made for the 203 ingredients using the integrated prediction system. Based on the mt-QSAR models, only compounds with positive results from both NB and RP algorithms were considered to be active on their target. The predictive results of NB and RP are given in Supplementary Tables 2 and 3. Based on CDOCKER models, compounds whose CE to one target was lower than the lowest CE of co-crystallized ligands were considered to be active on their target. Detailed information on the CDOCKER models is given in Supplementary Table 1. The number of predicted active compounds per target is shown in Figure 4A. The number of active compounds for M2, NMDAR, NF-κB, RT, GCPII, and CLK1 exceed the average of 29.8. The number of compounds acting on different numbers of targets is shown in Figure 4B. Finally, 28 compounds that act on three or more targets containing one or more viral targets were identified; the ligand-target interaction analysis is shown in Figure 4C. Twelve targets interacted with 28 compounds, of which, NF-κB, M2, NP, TNF-α interacted with more than 10 active compounds in the vs. prediction. The numbers of active compounds for NF-κB, M2, NP, and TNF-α were 19, 18, 13, and 11, respectively.

### Experimental Validation

The 28 predicted multitarget compounds were applied to *in vitro* validation studies, which verified their activity at the protein level via activity on NA and TNF-α, and their overall antiviral efficacy at the cellular level.

#### The NA Inhibition Activity of CYZH Compounds

NA is a relatively stable homotetramer present on the flu viral surface. It has been accepted as a classic anti-flu drug target and is involved in the release of flu viral progeny particles. Predictive results indicated that 4 of the 28 compounds have potential activity on NA. An NA inhibition assay was conducted for the 4 potentially active compounds; their IC_50_ values are shown in Table 4. The IC_50_ values of chlorogenic acid and orientin against NA of H1N1 and H3N2 were below 100 μM, showing that the NA model has a 50% hit rate and the two compounds have activities on viral NA. However, the activity against NA—resistant strains was not shown at this concentration.

**Table 4:** The evaluation of the activity of constituents from CYZH based on an NA inhibition test (μM).

**Compound**	**A/PR/8/34 (H1N1)**	**A/Minfang/15/90 (H3N2)**	**A/HebeiXinhua/** **SWL1106/2017**	**A/FujianXinluo/** **SWL2457/2014**
Chlorogenic acid	64.61 ± 6.97	57.15 ± 2.98	N/A	N/A
Orientin	53.71 ± 9.98	72.54 ± 2.97	N/A	N/A
Epigoitrin	N/A	N/A	N/A	N/A
Rupestonic acid	N/A	N/A	N/A	N/A
Zanamivir	0.0000176 ± 0.000013	0.000075 ± 0.000005	0.0000196 ± 0.0000074	0.0000913 ± 0.0000214

Data are expressed as mean ± SD (n = 3);

*N/A: IC_50_ > 100 μM*.

#### The Effects of CYZH Compounds on TNF-α Levels Determined by ELISA

**Figure 5:**
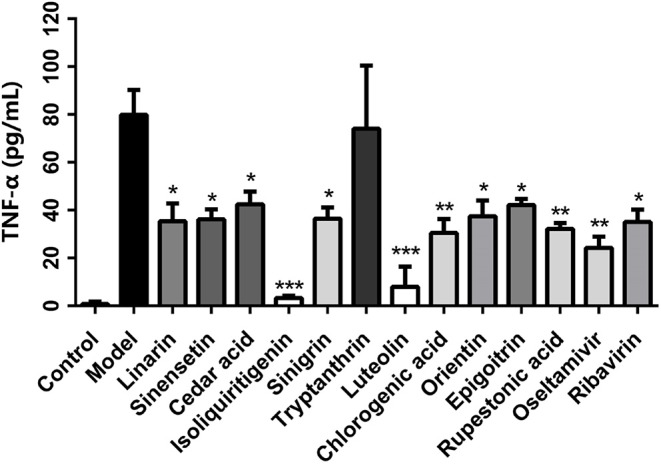
Effect of CYZH compounds on TNF-α expression in virus-infected A549 cells. Data are expressed as mean±SD (*n* = 3). *P < 0.05, **P < 0.01, ***P < 0.001 vs. Model group.

TNF-α can exacerbate inflammation and increase morbidity rates following flu infection, while neutralization of TNF-α can prolong survival by reducing pulmonary infiltration and lung injury. TNF-α expression in the cell culture supernatant of each group was measured by ELISA. Eleven of the 28 predicted compounds, linarin, sinensetin, cedar acid, isoliquiritigenin, sinigrin, tryptanthrin, luteolin, chlorogenic acid, orientin, epigoitrin, and rupestonic acid, were predicted by the vs. system to be active on TNF-α. As shown in Figure 5, in comparison with the model group, all compounds except tryptanthrin, were associated with significantly less TNF-α based on ELISA results. Among them, isoliquiritigenin and luteolin had a greater effect on TNF-α than positive drugs. Based on these results, the TNF-α predictive model showed a strong predictive capability with a hit rate of 10/11.

#### Activity Evaluation of Wild-Type and Resistant IAV Strains by Cytopathic Effect Assay

In order to evaluate the overall antiviral activity of the 28 predicted active compounds, their cytotoxic effects were examined in MDCK cells. The results show that the maximal non-toxic concentration (TC_0_) values for all compounds were >100 μM.

Cytopathic effect (CPE) reduction assays were performed with different IAV strains, including wild-type and drug-resistant IAV strains. The CPE assay results for the wild-type strain A/Puerto Rico/8/34 (H1N1) and A/Minfang/151/2000 (H3N2) are summarized in Table 5. Oseltamivir and ribavirin showed efficacy in the four modes of action tested for the two virus strains, with IC_50_ < 100 μM. Acacetin exhibited stronger antiviral activity on H1N1 than oseltamivir under the administration mode tested, therefore acacetin may have a stronger inhibitory effect on H1N1 invasion. When H1N1 virus was pre-incubated and treated simultaneously with the drug compounds, the IC_50_ values for quercetin, luteolin, emodin, and apigenin were lower than for oseltamivir or ribavirin, suggesting that they had an effect on reducing H1N1 viral activity or impairing viral adsorption.

**Table 5:** The activity evaluation of constituents from CYZH in wild strains-induced CPE reduction assay (μM).

	**A/PR/8/34 (H1N1)**				**A/Minfang/151/2000 (H3N2)**			
**Compound name**	**Drug administration after virus inoculation**	**Virus inoculation after drug administration**	**Pre-incubation of virus and drug**	**Simultaneous action of virus and drug**	**Drug administration after virus inoculation**	**Virus inoculation after drug administration**	**Pre-incubation of virus and drug**	**Simultaneous action of virus and drug**
Acacetin	62 ± 5.91	N/A	77.44 ± 25.7	N/A	N/A	72.54 ± 11.74	12.46 ± 5.46	20.46 ± 0.86
Indirubin	N/A	N/A	N/A	N/A	N/A	52.58 ± 27.42	N/A	N/A
Tryptanthrin	N/A	N/A	72.01 ± 23.57	N/A	N/A	76.33 ± 3.4	17.74 ± 1.74	31.83 ± 11.56
Quercetin	N/A	N/A	8.59 ± 1.34	5.41 ± 0.14	N/A	N/A	9.99 ± 2.84	18.33 ± 3.52
Luteolin	N/A	N/A	4.53 ± 0.92	7.08 ± 0.3	N/A	N/A	44.69 ± 19.99	N/A
Emodin	N/A	N/A	55.85 ± 9.8	6.53 ± 0.29	N/A	N/A	18.24 ± 1.02	55.86 ± 6
Apigenin	N/A	N/A	16.69 ± 1.77	9.06 ± 0.41	N/A	N/A	9.28 ± 2.03	15.84 ± 1
Oseltamivir	83.44 ± 15.6	11.41 ± 7.44	74.89 ± 20.7	4.45 ± 0.24	77.94 ± 22.1	54.45 ± 14.24	6.25 ± 2.6	8.47 ± 1.73
Ribavirin	16.1 ± 1.47	47.51 ± 7.94	10.4 ± 2.19	16.35 ± 2.95	22.51 ± 6.16	62.45 ± 16.24	9.18 ± 0.99	21.65 ± 0.97

Data are expressed as mean ± SD (n = 3);

*N/A: IC_50_ > 100 μM*.

Based on H3N2-induced CPE assay results, the IC_50_ values for apigenin, indirubin and tryptanthrin were similar to oseltamivir and ribavirin when they were administered to cells before viral infection. This suggests they could have potential antiviral prophylactic effects on cells. Acacetin, quercetin, and apigenin had stronger effects than oseltamivir or ribavirin on pre-incubation and simultaneous action modes with the H3N2 virus, possibly due to weakening of H3N2 activity or viral adsorption. Overall, acacetin, indirubin, tryptanthrin, quercetin, luteolin, emodin, and apigenin showed protective effects on cells based on the CPE reduction assay with two wild-type IAV strains.

The details of CPE reduction assay for cells infected with mutant A/HebeiXinhua/SWL1106/2017 (Oseltamivir & amantadine-resistant H1N1) and mutant A/FujianXinluo/SWL2457/2014 (Amantadine-resistant H1N1) are summarized in Table 6. In the dual-oseltamivir and amantadine-resistant H1N1 CPE assay, the IC_50_ value of ribavirin was 29.8–57.9 μM, while oseltamivir had no effect on results in the modes tested. The IC_50_ values for quercetin, luteolin, and apigenin were <100 μM in all modes except for drug administration after viral infection; quercetin and luteolin showed stronger efficacy than ribavirin. Results suggest that these four compounds could prevent or reduce viral activity, or impair the adsorption of oseltamivir and amantadine-resistant H1N1 on cells. Oseltamivir and ribavirin showed inhibitory activities in CPE reduction assay results when tested on amantadine-resistant strains. Compared with oseltamivir, the inhibitory efficacies of quercetin, luteolin, and apigenin were strong when they were administered after amantadine-resistant virus inoculation. In contrast to ribavirin, the activities of quercetin and apigenin were stronger in the prophylactic mode of viral infection after drug pre-administration. In general, quercetin, luteolin, and apigenin demonstrated inhibitory efficacy in the CPE reduction assay with resistant strain infection.

**Table 6:** The activity evaluation of constituents from CYZH in resistant strains-induced CPE reduction assay (μM).

	**A/HebeiXinhua/SWL1106/2017**				**A/FujianXinluo/SWL2457/2014**			
**Compound name**	**Drug administration after virus inoculation**	**Virus inoculation after drug administration**	**Pre-incubation of virus and drug**	**Simultaneous action of virus and drug**	**Drug administration after virus inoculation**	**Virus inoculation after drug administration**	**Pre-incubation of virus and drug**	**Simultaneous action of virus and drug**
Quercetin	N/A	19.1 ± 1.95	6.93 ± 4.6	14.59 ± 1.8	20.73 ± 14.71	86.1 ± 13.83	7.73 ± 3.04	37.05 ± 18.16
Luteolin	N/A	47.17 ± 7.14	15.22 ± 4.46	20.13 ± 10.56	31.86 ± 1.99	N/A	1.14 ± 1.02	24.95 ± 9.62
Apigenin	N/A	38.99 ± 3.82	31.63 ± 10.26	35.38 ± 3.98	28.12 ± 2.37	36.52 ± 5.79	32.11 ± 4.13	40.46 ± 4
Oseltamivir	N/A	N/A	N/A	N/A	86.87 ± 7.8	N/A	25.37 ± 8.32	25.5 ± 8.36
Ribavirin	57.9 ± 10.54	39.87 ± 9.71	29.8 ± 6.4	36.2 ± 13.7	25.94 ± 14.6	86.7 ± 9.8	24.64 ± 3.1	26.2 ± 2.4

Data are expressed as mean ± SD (n = 3);

*N/A: IC_50_ > 100 μM*.

### Gene Ontology Biological Process Analysis

**Figure 6:**
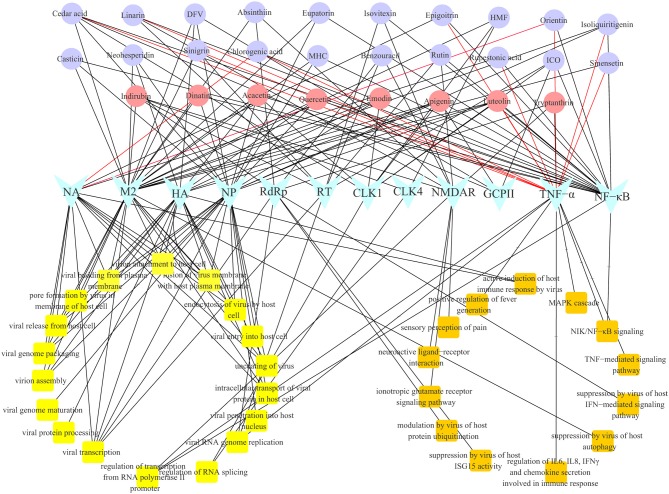
The network of compound-target-biological process analysis for CYZH.

Gene Ontology biological process (GO-BP) analysis was performed on the 28 multi-target compounds from CYZH to explore their possible mechanisms of action. The constructed compound-target-BP network is shown in Figure 6. Targets were involved in viral replication, and also in the immune response, inflammation, apoptosis and in neuroprotection, which are collectively termed NIM-related processes. Such processes include the MAPK cascade, the NF-κB signaling pathway, the TNF-mediated signaling pathway, the IFN-mediated signaling pathway, regulation of IL6, IL8, IFNγ, and chemokine secretion, regulation of host autophagy and ISG15 activity, the ionotropic glutamate receptor signaling pathway, regulation of pain, and fever generation. The results indicate that CYZH components act on multiple targets, whose action can directly affect IAV replication, and regulate immune and neuroprotective effects on the body.

## Discussion

Pandemics caused by IAV strains remain a serious threat to public health. Novel treatments are urgently required due to the high mutation rate of IAV and current drug-resistant strains. CYZH is an anti-flu medication, but information about its inhibitory mechanisms is not known. To uncover new agents against IAV, the 203 constituents of CYZH were collected and predicted using an *in silico* multi-target predictive system against IAV, which was constructed using a combination of NA, RP and CDOCKER methods. Twenty-eight compounds were acquired with activities predicted for three or more targets. Among them, rupestonic acid is the main active ingredient in *Artemisia rupestris L*. Previous studies had confirmed that it exhibited anti-influenza activity in obvious (Yong et al., 2013) or moderate (Obul et al., 2019) way. And Epigoitrin, a marker compound of *Radix isatidis*, was reported to exert antiviral activity against influenza virus FM1 by inhibiting virus attachment and proliferation *in vitro* (Xiao et al., 2016). These compounds are predicted to be against IAV by the vs. model constructed in this study.

To confirm the specific-target and multitarget profiles of the 28 compounds and the reliability of the vs. model, biological experiments were performed on single targets (NA and TNF-α) and their effects were assessed comprehensively at a cellular level. Based on NA inhibition assay results, chlorogenic acid and orientin had inhibitory activities against NA of wild influenza virus (IC_50_ < 100 μM). Chlorogenic acid and orientin were among the four chemicals predicted as potential NA inhibitors. However, they have less inhibitory activity against NA of resistant viruses than wild strains. Linarin, sinensetin, cedar acid, isoliquiritigenin, sinigrin, luteolin, chlorogenic acid, orientin, epigoitrin, and rupestonic acid exhibited significant effects on TNF-α expression; this was almost consistent with the predicted result. The study first discovered that rupestonic acid can target TNF-α, suggesting that it may regulate the expression of cytokines and chemokines by activating the TNF-α pathway to maintain the host's defense response to flu virus. We learn from these two experiments that chlorogenic acid and orientin are likely to be dual-targeted ligands against IAV at the very least. In the cellular experiment, acacetin, indirubin, tryptanthrin, quercetin, luteolin, emodin, and apigenin showed efficacies (IC_50_ < 100 μM) against the wild-type H1N1 strain and H3N2 strain. Quercetin, luteolin, and apigenin demonstrated protective effects (IC_50_ < 100 μM) for cells infected with the resistant IAV strains in CPE reduction assay. The two NA inhibitors, chlorogenic acid and orientin, showed no inhibition of virus-induced CPE in the concentration range of 100 μM. We suspected that their efficacies were not strong enough in NA inhibition assay so that they may be effective in CPE at concentrations above 100 μM. Results from GO-BP analysis of CYZH targets suggest that CYZH ingredients are involved in the pathways for viral replication, immune responses, inflammation, apoptosis, and neuroprotective processes.

In recent years, the study of multitarget-directed ligands has increased in popularity for the systemic discovery of novel drugs. Polypharmacology has emerged as a frontier cross-discipline, whereby effective drugs exert their therapeutic effect by modulating six targets on average (Anighoro et al., 2014). Compared to single-target drugs, agents targeted toward multiple proteins have greater efficacies and could circumvent drug resistance arising from single-target mutations or rare simultaneous mutations of several targets in different positions. Multitarget-directed studies could help toward finding multi-targeted drugs and may provide new indications or mechanisms of action for known drugs (Watanabe and Kawaoka, 2015). In our study, 15 targets, including viral proteins and host cellular proteins involved in the host respiratory system, nervous system and immune system, with functions in protecting the host against pathogens, blocking viral replication directly, and altering the biological network from a disordered state to a normal state, were taken for research.

Traditional Chinese medicines exert their therapeutic efficacy by targeting multiple proteins of the human body. Network pharmacology is an effective tool for establishing a “prescription - chemical composition - protein/gene - pathway - disease” network, which can be used to discover active ingredients or markers, and reveal the principles of drug combinations with the aid of computational processes (Zhang et al., 2019). In our study, to improve the reliability of multi-target prediction models, two machine learning algorithms, NB and RP, were used to build prediction models to predict CPIs for 12 targets. For the remaining three key targets without enough inhibitors/activators, molecular docking was used to explore their CPIs. This integrated prediction system is powerful for predicting the probability of interactions between a panel of c and targets.

Some limitations exist in the present study. Several novel-structure compounds with predicted activities were unobtainable, therefore their activities could not be verified. We believe that these will be acquired and verified in the future. In addition, to make the current model more powerful, active compounds should be continuously supplemented into training sets from online databases, literature, and experiments.

By combining a multi-target vs. method, pathway analysis, and experimental validation, it was revealed that the activity of CYZH against IAV infection occurs through compound groups interacting with multiple targets by blocking viral replication, and modulating host immune responses, inflammation, neuroprotection, autophagy, and apoptosis. Experimental results verified that chlorogenic acid and orientin in CYZH simultaneously targeted NA and TNF-α, which represent direct and indirect suppression of IAV. Compared to viral protein-targeted drugs, CYZH has advantages in limiting viral replication, regulating the body's steady state through immune system activation, and reducing host nerve discomfort or damage caused by flu by acting on neuroprotective targets. This study provides a theoretical basis for the development and clinical application of CYZH, and information for the discovery of anti-flu multitarget drugs. It was firstly reported that seven compounds were active in cellular experiments; of these, quercetin, luteolin, and apigenin demonstrated anti-resistant IAV activity. As promising broad-spectrum candidates, these compounds could be further studied for their *in vivo* efficacy, mechanisms, and structural transformations. Moreover, the high hit rates for compounds active toward NA, TNF-α, and IAV-induced cytopathic effects demonstrate that the constructed computational model has strong promise for screening active candidates within anti-flu TCMs in a highly efficient and cost-saving manner.

## Conclusion

In summary, NA, RP, and CDOCKER algorithms were used to construct a predictive system based on a series of flu-related viral and host targets that are involved in assisting viral replication and NIM-related processes. The application of the predictive system to the traditional Chinese medicine CYZH uncovered its active constituents and polypharmacological features. An experimental approach was used to validate predicted results, and several constituents were active on both wild-type and resistant IAVs. Combined with GO-BP analysis, the network action mechanism of CYZH was partially revealed. This study will lay an experimental foundation for the development of broad-spectrum antiviral drugs, and provide an efficient multi-target predictive tool for the discovery of new drugs against influenza.

## Data Availability Statement

All datasets generated for this study are included in the article/Supplementary Material.

## Author Contributions

AL contributed conception and design of the study. LX did experiments and wrote the first draft of the manuscript. WJ performed the statistical analysis. HJ, LZ, and JX wrote sections of the manuscript. AL and GD modified the draft of the manuscript. All authors contributed to manuscript revision, read, and approved the submitted version.

### Conflict of Interest

The authors declare that the research was conducted in the absence of any commercial or financial relationships that could be construed as a potential conflict of interest.
